# Ruptured Left Gastric Artery Aneurysm Successfully Treated by Thrombin Injection: Case Report and Literature Review

**DOI:** 10.1100/tsw.2005.8

**Published:** 2005-01-21

**Authors:** S. Chandran, A. Parvaiz, A. Karim, I. Ghafoor, B. Steadman, N. W. Pearce, J.N. Primrose

**Affiliations:** Department of Surgery, Southampton University Hospital, Southampton, UK

**Keywords:** gastric artery, aneurysm, rupture, endovascular, percutaneous, embolisation

## Abstract

This short report describes the successful use of a new minimally invasive technique for the treatment of acute gastric artery aneurysm rupture. It emphasises the importance of persistence and multiple imaging modalities in the presence of gastrointestinal bleeding. The photographs and case history clearly illustrate the nonoperative management and highlight learning points for experienced surgeons and trainees alike in the management of this potentially fatal condition.

## INTRODUCTION

Aneurysms of the gastric and gastroepiploic arteries account for only about 4% of all splanchnic artery aneurysms[1]. However, rupture is associated with a mortality of up to 70%[2] and usually warrants urgent surgical intervention. We present an interesting case of a patient who presented with haematemasis following rupture of a left gastric artery aneurysm that was successfully treated by percutaneous thrombin injections. A review of the literature for this rare condition is also presented.

## CASE REPORT

A 59-year-old lady was transferred from a district general hospital with a history of acute upper abdominal pain and haematemesis. Her past medical history included elective embolisation of a latent gastroduedenal aneurysm 3 years previously, surgery for pancreatic pseudocyst, depression, and alcohol abuse. On admission, she was resuscitated and underwent urgent endoscopy; a small superficial duodenal ulcer was identified and injected successfully. The following day, she developed melaena, hypotension, and anaemia that required further blood transfusion and aggressive fluid therapy. A repeat endoscopy was therefore arranged and demonstrated blood in the stomach and duodenum, but with no obvious source.

Further episodes of hypotension and haematemasis that day prompted an urgent mesenteric angiogram and a leaking pseudoaneurysm arising from the left gastric artery was identified (Fig. 1). Under computerised tomography (CT) guidance, a 21-gauge Chiba needle was introduced into the sac percutaneously (Fig. 2) and the aneurysm was embolised with thrombin injections. A repeat angiogram 2 days later (Figs. 3 and 4) confirmed the successful occlusion. The patient went on to make a good recovery and was discharged home shortly afterwards. She has been followed up as an outpatient and remains asymptomatic at 12 months.

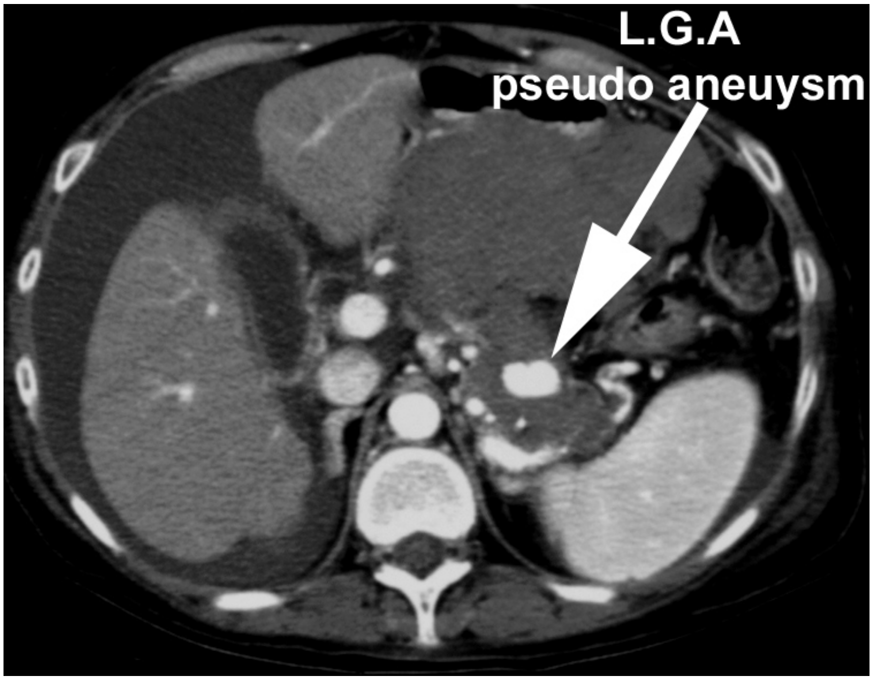

**FIGURE 1**. Late arterial phase CT demonstrating aneurysm anterior to the splenic artery. A large anterior haematoma is noted within the lesser sac with a moderate volume haemoperitoneum.

## DISCUSSION

Splanchnic artery aneurysms are rare. However, the increasing availability of ultrasound scan, CT, and subtraction angiography is resulting in the increased identification of these pathologies. Of the identified lesions, the vast majority occur in the splenic artery with gastric and gastroepiploic artery aneurysms accounting for only 4%[1]. Gastric artery aneurysms are three times more common in males and the vast majority are identified in people over 50 years of age[3]. Gastric artery aneurysms are usually solitary lesions occurring as a result of periarterial inflammation or medial degeneration. Arteriosclerosis, when present, is thought to be secondary and not a cause[4]. Aneurysms have also been identified following and described as a complication of acute pancreatitis[5]. With a rupture rate of 90%[2], the commonest clinical presentation of gastric artery aneurysm is as a surgical emergency with epigastic pain and hypovolaemic shock. The majority of lesions are intramural (70%) and present with torrential haematemasis, the remaining extramural ruptures presenting with intraperitoneal haemorrhage[6]. With a mortality of 70%[2], aggressive fluid resuscitation is mandatory while urgent ligation, excision, or embolisation by open, laparscopic, or interventional techniques is necessary to control the life-threatening bleeding[8]. Due to this high rate of rupture and subsequent mortality, all patients diagnosed with incidental gastric artery aneurysms should be electively ligated or embolised to avoid the risks of subsequent rupture[9].

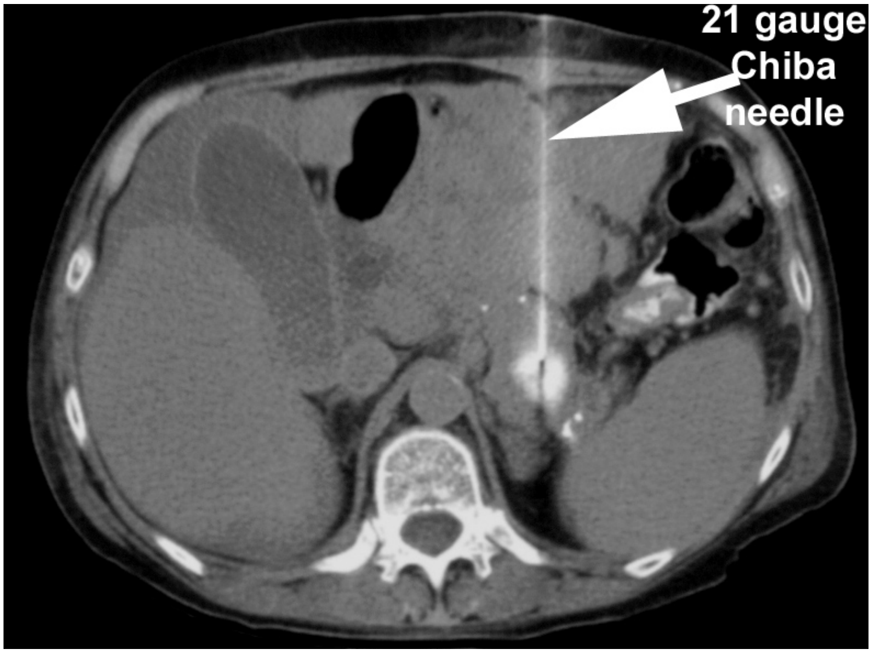

**FIGURE 2.** Under CT guidance, a 21-gauge Chiba needle has been introduced into the aneurysm and dilute contrast with thrombin slowly injected.

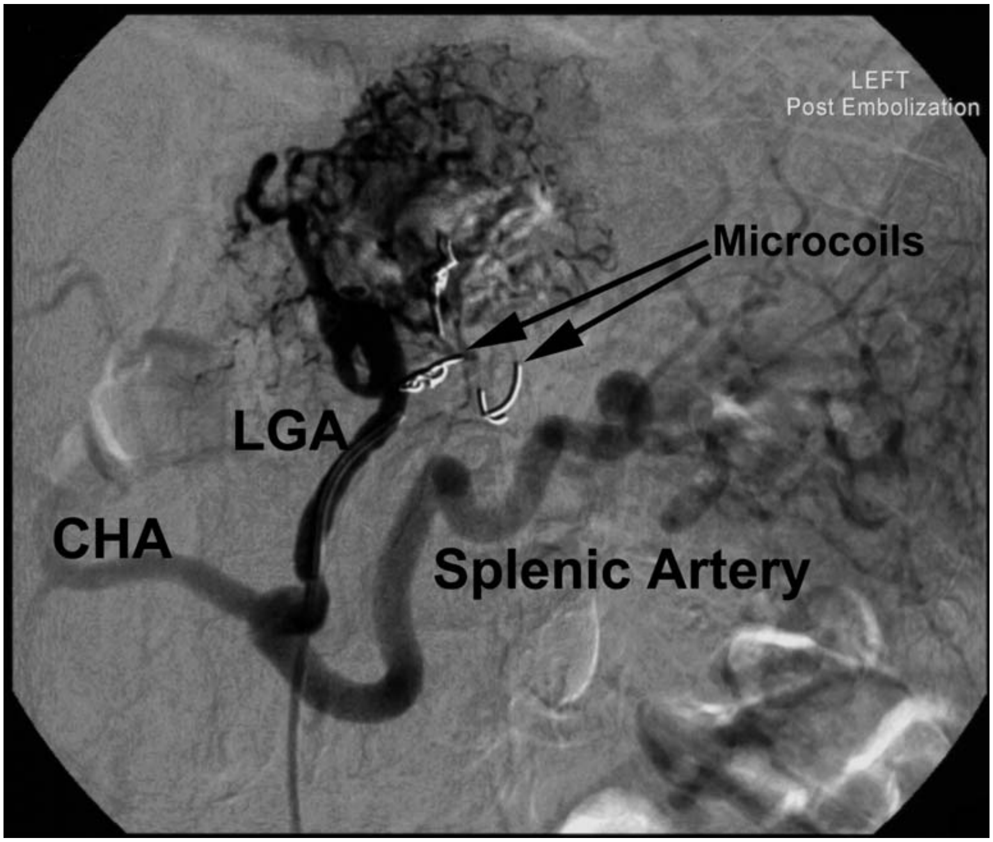

**FIGURE 3**. The left gastric artery has been selectively catheterised with a Tempo 4 C2 catheter and a coaxial microcatheter used to assess the feeding artery of the aneurysm.

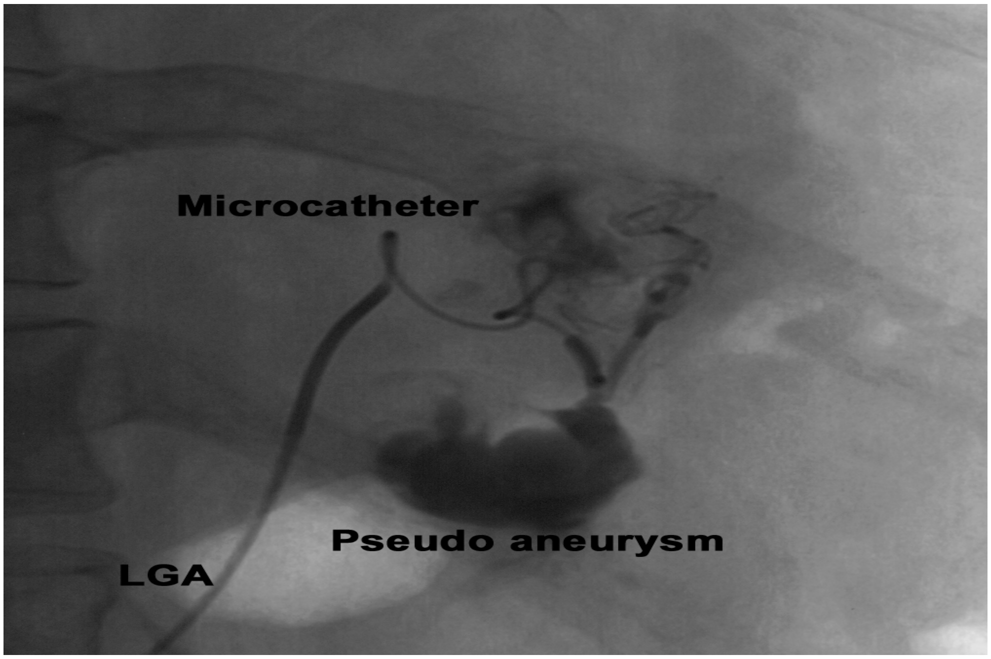

**FIGURE 4**. Completion celiac axis angiography demonstrates successful occlusion of the aneurysm.
